# Indiana Medical Society, Held at Indianapolis, May 18, 1870

**Published:** 1870-06

**Authors:** 


### INDIANA MEDICAL SOCIETY.

INDIANAPOLIS, Ind., May 18.

The Indiana Medical Society concluded its session to-day. All persons desiring to be members were requested to pay $2 before participating in the proceedings. Various papers were read upon medical questions. The following officers were elected: President, Dr. R. N. Todd; Vice President, J. N. Rosenthall, of Fort Wayne; Secretary, G. V. Woolen; Assistant Secretary, Dr. W. J. Elstun; Treasurer, J. H. Woodburn; Librarian, A. W. Davis. A committee of one from each congressional district was appointed to collect and collate facts relating to the health of their different localities, medical statistics, etc. Delegates to the American Medical Association were appointed. The next meeting of the society was fixed for the second Tuesday in June next. The President appointed the standing and special committees for the ensuing year. Adjourned.

